# Genetic interactions in a *C. elegans sod-1* ALS model: glutamatergic neuron degeneration

**DOI:** 10.17912/micropub.biology.000338

**Published:** 2021-01-15

**Authors:** Jennifer F Osborne, Katherine S Yanagi, Anne C Hart

**Affiliations:** 1 Department of Neuroscience, Brown University, Providence, RI 02912; 2 Robert J. and Nancy D. Carney Institute for Brain Science, Providence, RI 02912; 3 Neuroscience Graduate Program, Brown University, Providence, RI 02912

**Figure 1. Assessment of genetic interactions:**
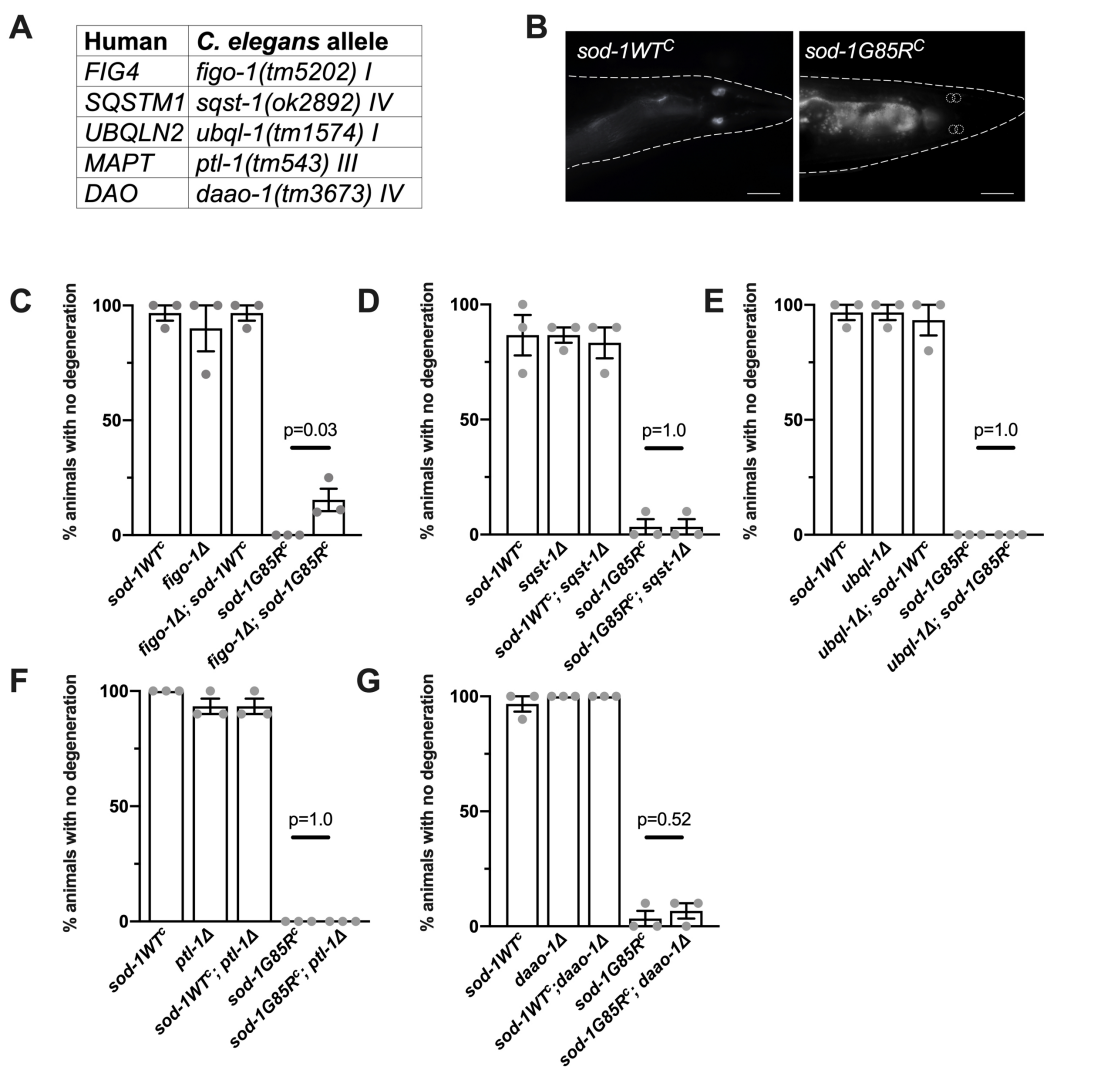
(A) ALS genes of interest (left) and corresponding deletion alleles in *C. elegans* orthologs (right). (B) Representative images from *sod-1WT^c^* (left) and *sod-1G85R^c^* (right) after 22 hours of paraquat exposure. Animals with three or four phasmid neurons that fill with dye are scored as having no degeneration.Animals are outlined, fluorescence in the gut is visible in each image, and location of missing neurons is outlined in *sod-1G85R^c^*. 63x magnification,scale bar 20µm. (C) Glutamatergic neuron degeneration: after 22 hours of paraquat exposure, an increased percentage of *figo-1(tm5202); sod-1G85R^c^* double mutant animals showed no degeneration, compared to *sod-1G85R^c^* animals. Three independent trials totalling n=30 animals, two-tailed t-test, p=0.03. Dots represent the average from each trial of 10 animals. Error bars indicate standard error of the mean. All data were collected blind to genotype. (D-G) Glutamatergic neuron degeneration as described in C for other genotypes.

## Description

Amyotrophic lateral sclerosis (ALS) is a fatal degenerative motor neuron disease. While the mechanisms underlying motor neuron death in ALS are not well understood, mutations in over 25 genes can cause this disease (Marangi and Traynor 2015). It remains unclear which, if any, of these genes act in the same disease-associated pathway(s), or if they act in the same pathway(s) as genes associated with the related disorder, frontotemporal dementia (FTD) (Ling *et al.* 2013). The first ALS-causing gene to be identified was superoxide dismutase 1 (*SOD1*), a regulator of cytoplasmic redox homeostasis (Rosen *et al.* 1993). We can begin to construct a pathway for neurodegeneration through *SOD1* by identifying genes whose loss of function (LOF) modifies the level of degeneration in a *C. elegans SOD1* ALS model. This will contribute to our understanding of whether ALS/FTD genes act in a single or multiple pathways to cause disease.

To undertake this analysis, we introduced LOF alleles for *C. elegans* orthologs of ALS or FTD genes into a *C. elegans* knock-in model of *SOD1* ALS (Baskoylu *et al.* 2018). Previously, we used CRISPR/Cas9 to introduce a point mutation corresponding to the ALS-causing *SOD1 G85R* variant (Rosen *et al.* 1993) into a conserved residue in the endogenous *C. elegans sod-1* gene (Baskoylu *et al.* 2018). *sod-1(rt448)* contains the G85R mutation and *sod-1(rt449)* is the corresponding control strain containing silent edits; these are referred to herein using the corresponding human patient allele nomenclature, *sod-1G85R^C^* and *sod-1WT^C^* (Baskoylu *et al.* 2018). *sod-1G85R^C^* animals exhibit glutamatergic neuron degeneration following exposure to mild oxidative stress. To begin exploring genetic interactions in this model, we selected several genes with existing deletion alleles (Figure 1A); deletion alleles were crossed into the *sod-1G85R^C^* background to investigate their effect on *sod-1G85R* glutamatergic neuron degeneration. ALS/FTD-causing gene orthologs tested include: *figo-1,* ortholog of factor-induced gene 4 (*FIG4*), a regulator of PI(3,5)P_2_, a phosphoinositide phosphatase involved in endosomal signaling and trafficking (Chow *et al.* 2009); *sqst-1,* an ortholog of human sequestosome 1 (*SQSTM1*)*,* which encodes the SQSTM1/p62 cargo protein involved in selective autophagy (Fecto *et al.* 2011); *ubql-1*, ortholog of ubiquilin-2 (*UBQLN2*), a carrier in the ubiquitin/proteasome system (Deng *et al.* 2011); *ptl-1,* ortholog of microtubule-associated protein tau (*MAPT*), a player in microtubule assembly and dynamics also implicated in Alzheimer’s disease and various dementias (Fang *et al.* 2013, Rademakers *et al.* 2004); and *daao-1*, ortholog of D-amino acid oxidase (*DAO*), among whose substrates is D-serine, an endogenous neurotransmitter and co-agonist of NMDA receptors (Mitchell *et al.* 2010).

Loss of glutamatergic neurons was quantified by dye-filling four glutamatergic neurons (PHAR/L and PHBR/L) in the tail with exposed sensory endings (Figure 1B). After mild oxidative stress with paraquat (22 hours, 2.5mM), the majority of these neurons in *sod-1G85R^C^* animals are unable to take up dye and are presumed to have degenerated (Baskoylu *et al.* 2018). *figo-1(tm5202); sod-1G85R^C^* animals exhibited increased dye-filling compared to *sod-1G85R^C^* (Figure 1C), indicating loss of *figo-1* partially suppresses glutamatergic neuron degeneration in *sod-1G85R^C ^*animals. *sod-1G85R^C^; sqst-1(ok2892), ubql-1(tm1574); sod-1G85R^C^,* *sod-1G85R^C^; ptl-1(tm543),* and *sod-1G85R^C^; daao-1(tm3673)* animals exhibited no significant difference in dye-filling compared to *sod-1G85R^C^* (Figure 1D-G). LOF alleles did not show stress-induced neuron degeneration in combination with *sod-1WT^C^* (Figure 1C-G).

Based on these results, *figo-1* and *sod-1* may lie in the same genetic pathway. *sod-1* LOF is predominantly responsible for glutamatergic neuron degeneration in *sod-1G85R^C^*animals (Baskoylu *et al.* 2018). As *figo-1* LOF suppressed glutamatergic neuron degeneration in the *sod-1G85R^C^*model, *figo-1* likely lies downstream of *sod-1*. The epistatic relationship observed here will need to be confirmed with other deletion alleles of *figo-1* before potential mechanisms of *FIG4* and *SOD1* interaction are investigated. *figo-1* may make neurons more robust or specifically resistant to *sod-1*-related glutamatergic neuron loss. Additionally, *SOD1* ALS-related alterations in endosomal activity have been reported (van Dis *et al.* 2014; Xie *et al.* 2015) and may be influenced by the indirect role of *FIG4* in endosomal signalling and trafficking. Further examination of epistatic relationships between *SOD1* and other ALS/FTD-causing genes can yield a complete pathway of *SOD1* action, as well as elucidate whether ALS/FTD is a single disease consisting of one or more pathways or perhaps even multiple diseases with shared characteristics.

## Methods

***C. elegans* strains**

All strains were maintained using standard methods (Brenner 1974) and constructed using the *C. elegans* Bristol variety N2 as a wild-type parent strain. All strains were backcrossed to N2 at least 4 times before use.

List of *C. elegans* strains (Strain name: *genotype*)

• HA2986: *sod-1(rt448[WTC]) II; pha-1(+) III* (abbreviated *sod-1WT^C^*)

• HA3299: *sod-1(rt449[G85RC]) II; pha-1(+) III* (abbreviated *sod-1G85R^C^*)

• HA3261: *figo-1(tm5202) I*

• HA3266: *sqst-1(ok2892) IV*

• HA3352: *ubql-1(tm1574) I*

• HA3402: *ptl-1(tm543) III*

• HA3361: *daao-1(tm3673) IV*

• HA3788: *figo-1(tm5202) I; sod-1WT^C^ II*

• HA3789: *figo-1(tm5202) I; sod-1G85R^C^ II*

• HA3509: *sod-1WT^C^ II; sqst-1(ok2892) IV*

• HA3510: *sod-1G85R^C^ II; sqst-1(ok2892) IV*

• HA3516: *ubql-1(tm1574) I; sod-1WT^C^ II*

• HA3517: *ubql-1(tm1574) I; sod-1G85R^C^ II*

• HA3996: *sod-1WT^C^ II; ptl-1(tm543) III*

• HA3997: *sod-1G85R^C^ II; ptl-1(tm543) III*

• HA3795: *sod-1WT^C^ II; daao-1(tm3673) IV*

• HA3796: *sod-1G85R^C^ II; daao-1(tm3673) IV*

• FX19472: *tmIn10 [mIs14 spy-10(e128)] II*, used in strain construction

Strains are available upon request to anne_hart@brown.edu.

**Dye-filling assay**

To quantify loss of glutamatergic sensory neurons, we followed a procedure outlined in Perkins *et al.* 1986. Animals raised to larval stage 4 were transferred to plates containing 2.5 mM paraquat (Acros Organic, #1910-42) for 22 hours at 25C with minimal light exposure. After the incubation period, 2 mg/mL DiI (Fisher DiIC18(5) D307) was added. After 1.5 hours, animals were transferred back to regular NGM plates, then immobilized with 30 mg/mL 2-3-butanedione monoxime (BDM, Sigma) in M9 buffer and mounted on 2% (vol/vol) agar pads. Fluorescent neuronal cell bodies in the tail were counted under 63x objective on a Zeiss Axioplan2. Animals with three or four phasmid neurons that fill with dye were scored as having no degeneration (Figure 1B).
